# Nasal Reconstruction of Post-Mohs Defects >1.5 cm in a Single Cosmetic Subunit Under Local Anesthesia by a Combination of Plastic Surgeon and Mohs Surgeon Team: A Cross-sectional Study and Review of Algorithmic Nasal Defect Closures

**DOI:** 10.1097/GOX.0000000000002277

**Published:** 2019-06-05

**Authors:** Omeed M. Memar, Benjamin Caughlin

**Affiliations:** From the * Academic Dermatology & Skin Cancer Institute, Chicago, Ill.; † Department of Surgery, Division of Otolaryngology, John H. Stroger, Jr. Hospital of Cook County, Chicago, Ill.; ‡ Division of Facial Plastic and Reconstructive Surgery, Jesse Brown VAMC, Chicago, Ill.; § Division of Facial Plastic and Reconstructive Surgery, University of Illinois Health Hospital System, Chicago, Ill.; ¶ Kovak Cosmetic Center, Oakbrook Terrace, Ill.

## Abstract

**Introduction:**

We present an algorithmic approach to the reconstruction of larger post-Mohs defects treated in a practice with both a plastic surgeon and Mohs surgeon. The aim of the study is to present post-Mohs reconstructive choices made by our team compared with closures done by solo dermatologists.

**Methods:**

A cross-sectional study was designed. Participants were 66 consecutive cases of nasal Mohs repairs performed under local anesthesia. For each Mohs case, data were collected on the age of the patient, smoking status, tumor type, tumor location, tumor diameter, the number of Mohs stages needed to clear the surgical margins of any remaining cancer, final defect diameter, reconstructive methods used, and postoperative complications.

**Results:**

Basal cell carcinoma was the most common tumor treated on the nose, and post-Mohs basal cell carcinoma defects were closed predominantly with full-thickness skin grafts (FTSGs), rotation flap (RF), or a combination of both. The sidewall was the cosmetic unit most affected by skin cancer, and defects were commonly closed by FTSG and RF. Fifty percent of the sidewall defects required more than 1 closure method, compared with 24% of the nasal tip defects. FTSG combination closure was performed on 20 cases, usually with an advancement flap.

**Conclusions:**

The addition of a plastic surgeon shifted the nasal reconstructive techniques when compared with dermatologists alone. Post-Mohs defect >1.5 cm in a single cosmetic subunit was reconstructed under local anesthesia with either RF or a combination of FTSG and an advancement flap, whereas dermatologists most commonly chose a primary closure.

## INTRODUCTION

MMS was invented by Dr. Frederic Mohs and has proven to be a very accurate method of resecting contiguous tumors.^1^ The difference between MMS, and excision that is sent to a pathologist as a frozen section, is in the way the specimen is examined. With MMS, 100% of the deep and peripheral margins are examined.^2^ However, a frozen section sent to a pathologist is converted into bread-loaf slices; statistical studies show that the pathologist examines around 0.1% of the true margins and extrapolates the rest.^3^ Tumor 5-year recurrence rates are significantly lower for primary basal cell carcinoma (BCC) treated by MMS compared with excision with margins, radiotherapy, or curettage.^4^ SCC^5^ and melanoma in situ^6^ treated by MMS versus excision have 5-year recurrence rates that favor MMS. Seidler et al^7^ showed that MMS has a superior patient quality-adjusted life years and cost-effectiveness compared with traditional excision. Recent transcutaneous imaging techniques have attempted to reproduce the accuracy of BCC detection in vivo, but the technology is not a standard of care.^8,9^

We have previously shown that a combination of an MS and PS is an ideal combination: most accurate resection method for contiguous tumors, a wider range of closure techniques by a plastic surgeon, convenience for the patient, and avoidance of general anesthesia.^6,7,10^ However, after SC removal using MMS, the majority of defects are reconstructed by dermatologists.^12^ The PS is predominantly a referral source from a Mohs surgeon.^11^

Because the nose is the most common location for BCC,^12^ we decided to study our nasal defect data. Nasal reconstruction was developed >2,000 years ago in India. Subsequently, great figures perfected the art of nasal reconstruction.^13^ Nasal cosmetic subunits were proposed in 1985.^14^ Elements of nasal reconstruction include maximal conservation of normal tissue, reconstruction of the defect and not the subunit, complementary ablative procedures, primary defatting of full-thickness skin grafts (FTSGs), the use of axial pattern flaps, and focus on contour as the endpoint of reconstruction.^15^ The approach to nasal reconstruction has been presented algorithmically.^16^ We modified previous algorithms by combining reconstructive methods. We found that our choices allowed for the reconstruction of larger nasal defects under local anesthesia.

The aim of our study is to present a large number of nasal post-Mohs defect (PMD) that were closed under local anesthesia with a multidisciplinary team. We compare our results with other Mohs surgeons who do not use plastic surgeons.^5^

## METHODS

### Patient Selection

The criteria for referral to MMS is based on established recommendations.^17^ None of the patients in this group had neural/vascular invasion. All SCC lesions were well differentiated and <2 cm in lesional diameter. Melanoma was excised with 0.5-cm margin for diagnosis/staging; MMS was used to resect any remnant cells.

Sixty-six post-Mohs nasal defects, from July 1, 2016, to July 1, 2017, were studied. Local anesthesia was used.^18^ For each MMS, the following data were collected: patient age, smoking status, tumor type, nasal subunit involved, tumor diameter, the number of Mohs stages needed to clear the margins, PMD diameter, complications, and the reconstructive methods used. Inquiry was made of patient satisfaction with the reconstruction upon follow-up. Revisions were made, including scar revision, resurfacing, fat transfer, and steroid injections, until all patients were satisfied with their end results. All patients were satisfied after the final treatment.

### Data Analysis

The statistical data were completed with the analysis of variance being used to test if the number of Mohs stages needed to clear the defect was associated with the different repair options; a *P* value was determined based on the association of nasal defect locations and closure options selected.^6^

## RESULTS

Of the 66 participants, 48 participants were nonsmokers who had never smoked, 4 participants were nonsmokers who quit in the past 1–5 years, 11 participants were nonsmokers who quit ≥10 years prior surgery, and 3 participants were smokers. Our analysis showed that the number of Mohs stages associated with different repairs was statistically different (analysis of variance, *P* < 0.001). The descriptive numbers (Table 1) show that the average age of patients requiring more than one closure method was higher among female patients. Female patients had larger tumors, whereas patients requiring more than one closure method had the greatest number of Mohs stages required to clear margins. Patients requiring more than one closure method had the largest PMD.

**Table 1.**
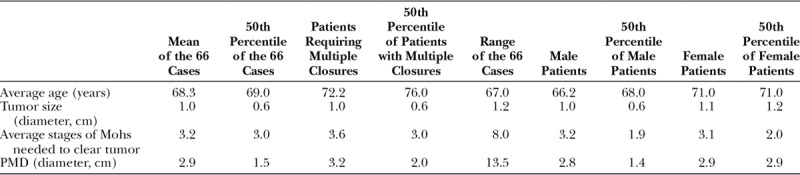
Demographics of Patients, Including Age, Tumor Size, Mohs Stages, PMD Size Compared with the Mean Number of Cases, the Number of Cases Requiring Multiple Closures, and the Sex of the Patients

Analysis of the descriptive data shows that BCC was the most common diagnosis warranting MMS on the nose (Table 2). FTSG was the most common closure for BCC on the nose, whereas the rotation flap (RF) was the most common closure when all tumor types were combined. None of the 3 smokers had an FTSG placed: 1 patient had a linear closure, another patient had an advancement flap (AF), and yet another patient had an RF; all flaps survived without complication.

**Table 2.**
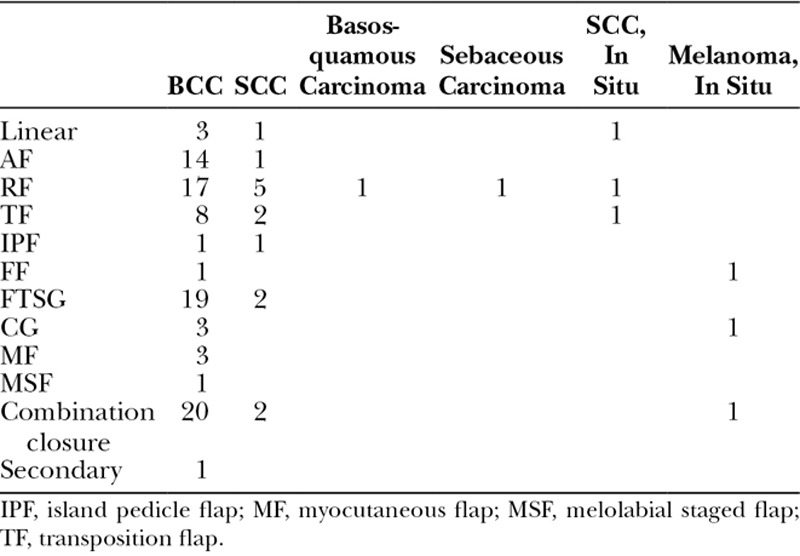
Total Number of Closure Methods Based on Tumor Diagnosis

The nasal sidewall was the site of most SCs (Table 3). The nasal sidewall defects were commonly reconstructed using an RF followed by an FTSG.

**Table 3.**
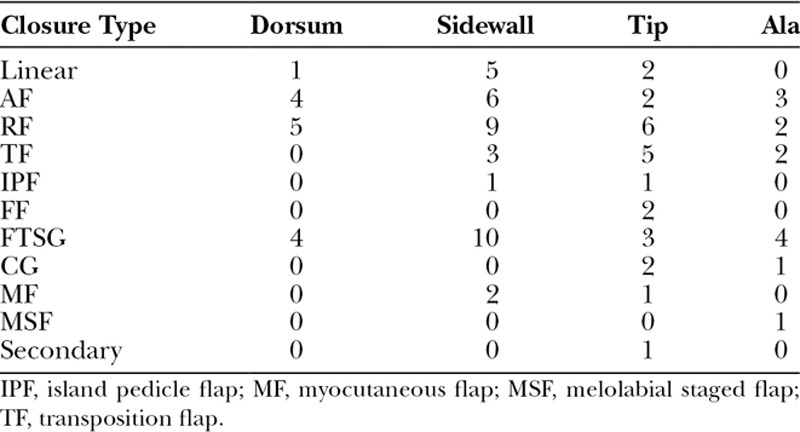
Number of Repairs Associated With Each Closure Type at Different Areas of the Nose

As a combined cosmetic unit, both sidewalls were most affected by SC, with the left side greater than the right side (Table 4). Men were more often affected on the tip, and women were more often affected on the sidewall. The sidewall commonly required multiple closure techniques. The dorsum required multiple repairs in men but not in women.

**Table 4.**
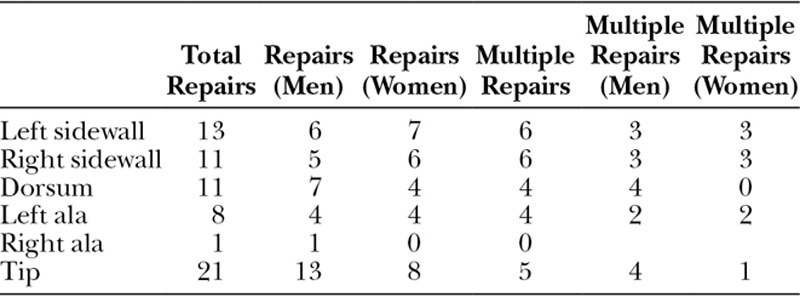
Defects of Cosmetic Subunits in Men, Women, and Defects Requiring Multiple Repairs

Only 11 cases had postoperative complications, with 9 cases of hypertrophic scarring or depressed scarring. The scarring was improved using cortisone injections, fractional resurfacing, or fat transfer. One case developed a pyogenic granuloma that was excised to rule out tumor recurrence. Three cysts were drained. One-week postoperative, 64 of 66 repairs were reported as a good result, by patients, not requiring further refinement. Two patients, both with interpolation flaps, were not happy with the appearance 1 week after the final sutures were removed. After repeated cortisone injections, fat transfer to depressions, and scar revision surgery, both patients were happy at 1-year postoperative (Fig. 4).

Of the 21 FTSGs performed on the nose, 20 FTSGs were in combination with flap closures (Table 5). Ten of the 20 FTSG combinations were on the sidewall.

**Table 5.**
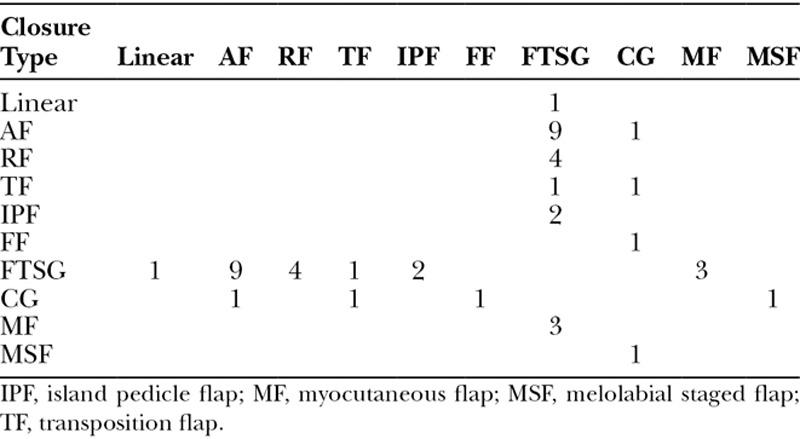
Multiple Closure Combinations

## DISCUSSION

This study presents the benefits of MSs and PSs working together. Currently, MSs reconstruct 83.9% of nasal PMDs, with primary repair as the predominant reconstruction.^5^ Our study reveals that the presence of a PS increases the variety of closure options. The ideal closure of a PMD is the simplest and most cost-effective measure that reestablishes cosmesis and function.

Yearly, around 876,000 MMSs are performed in the United States and the number is rising.^19^ Only 16.1% of PMDs are referred out to nondermatologists for reconstruction.^20^ We show that the team approach of MS/PS exposes the PS to 100% of the PMD and benefits the patient with a different reconstructive approach. PMDs ≤1.5 cm were more common on the ala, followed by the tip,^21^ whereas we demonstrated that defects >1.5 cm were more common on the sidewall followed by the tip. A larger study of PMDs revealed that the majority of defects were on the dorsum and sidewalls; however, they failed to report on the PMD size.^22^ We found that the ala was the least common site for defects >1.5 cm. Although MSs did not report the use of cartilage graft (CG), PSs used CGs, especially for the ala. We used CGs mainly for tip and alar defect. We used FTSG in combination with AF or RF, especially on the sidewall. We chose to combine multiple closure methods, whereas others treated 29.8% of nasal PMD >1.5 cm with forehead flap (FF).^23^

Because each defect is different in shape, site, and size, an algorithmic approach was undertaken to place closures in outlines of cosmetic subunits, while performing the procedure under local anesthesia (Fig. 2).

**Fig. 1.**
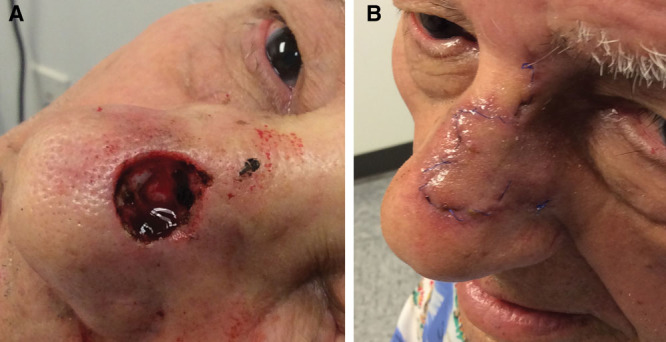
Mohs excision of BCC on the dorsum, repair with a bilobed flap. A, Post-Mohs resection of BCC on right NS. B, Postbilobed TF closure, borrowing excess skin superiorly. NS, nasal sidewall; TF, transposition flap.

**Fig. 2.**
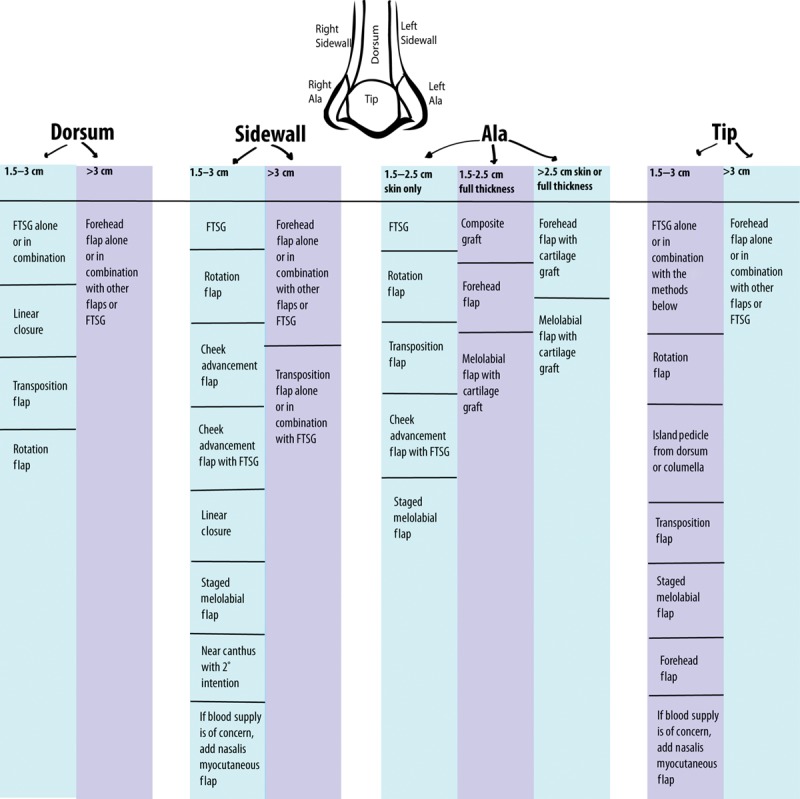
Algorithmic approach to the closure of post-Mohs nasal defects ≥1.5 cm wide per individual cosmetic subunits under local anesthesia. The intention of the algorithm is to determine the location and size of defect and then select the closure options listed that would place closures along the borders of cosmetic subunits.

Our approach to reconstructing PMDs >1.5 cm was algorithmic, per cosmetic units of the nose, the size of the defect, and a visual assessment of methods that align closures along known cosmetic subunits. Defects on the dorsum from 1.5 to 3 cm were reconstructed with an RF, preferably a Rieger flap (Figs. 1, 2). For areas requiring shadows, like the supratip, a fine strip of FTSG was added. The dorsum defects were made smaller by AF of the sidewalls, using bilateral cheek AF. For defects on the dorsum >3 cm, an FF was considered.

**Fig. 3.**
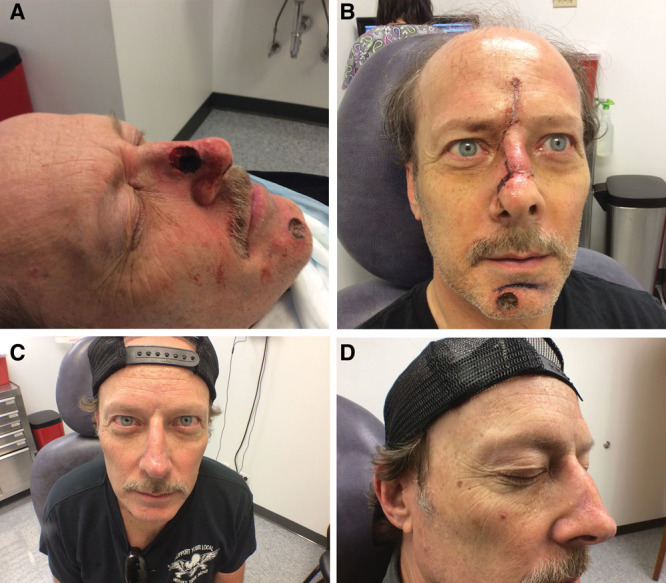
Mohs excision of BCC on the right nasal side wall, repair with a combination RF and cheek AF, and further revision with nonablative fractional 1,540 nm erbium glass laser. A, Post-Mohs resection of BCC on right NS. B, Post-Rieger RF and right cheek AF. C, After nonablative fractional 1,540 nm erbium glass laser, frontal. D, After nonablative fractional 1,540 nm erbium glass laser, ¾ view. NS, nasal sidewall.

For SD (1.5–3 cm), an RF was the preferred technique and FTSG was placed in areas of shadow, like the alar groove, or medial canthus. Fascial flaps released wound tension. For defects measuring >3 cm, either a combination of closures or FF was considered. Nasalis myocutaneous flap was used in avascular beds (Figs. 2, 3).

**Fig. 4.**
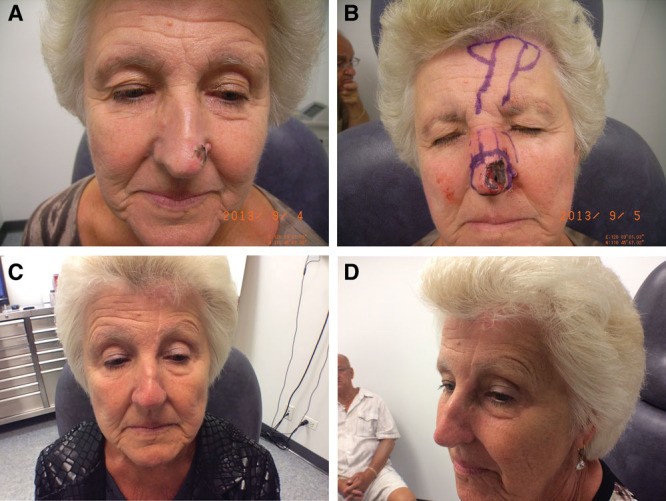
Mohs excision of melanoma in situ of thee nasal tip and ala with repair using a paramedian FF and further revision with fat grafting and nonablative fractional 1,540 nm erbium glass laser. A, Postexcisional biopsy of melanoma in situ with FTSG coverage and pre-Mohs on left nasal tip. B, Design of paramedian FF closure and mucosal AF. C, Postseparation of the pedicle; however, there is a supratip groove. D, Postfat transfer to the supranasal groove and nonablative resurfacing with the 1,540 nm erbium glass laser.

Nasal tip defects (1.5–3 cm) were preferentially reconstructed with an RF. Defects >3 cm, an FF was considered. Most of our cases were skin deep; however, the full-thickness defects required a 3-layered approach: reestablishing mucosa, giving cartilaginous support, and a final vascularized flap (Figs. 2, 4).

For alar defects (0.5–2.5 cm), skin deep, and with adequate integrity, an FTSG was used alone or in combination with a sidewall AF. If alar valve integrity was compromised or a full-thickness defect was created, then a conchal CG was added. The skin was covered either with a transposition flap or with a melolabial staged flap. In select cases, an auricular composite graft was used. Larger defects of the ala required an FF (Figs. 2, 4).^24^

Postoperative complications were scarring, cysts, and pyogenic granuloma. Hypertrophic scarring was mostly seen in Fitzpatrick skin type V–VI and defects under tension. All hypertrophic scarring was improved to patient satisfaction with cortisone injections. Other scars were improved upon with a combination of fat grafting, laser resurfacing, and cortisone injections. Patients who smoked were reconstructed with local well-vascularized flaps and not FTSG. One-year tumor recurrences were not identified in the 66 cases, and all patients were happy with the final cosmetic results. FF was selected judiciously after discussing options with patients, including the forehead defect, the 3 weeks of interpolated flap connection, the subsequent bisection, and repairs. Given a choice, most patients did not choose the FF in our practice.

This is the first publication presenting a large number of nasal PMD >1.5 cm in diameter in a single cosmetic subunit that were reconstructed by a combined team of an MS and a PS, using local anesthesia. Our practice exposed the PS to almost 100% of the PMDs, as opposed to the national 16.1%.^5^ Because the use of MMS is growing in the United States and around the world, PSs would be well suited to establish a relationship with local MS. The significance of our data shows that the addition of an MS exposes the PS to many more SC excision defects, and the patients are offered a broader range of closure techniques done under local anesthesia. MMS with same-day reconstruction is convenient for patients and affords them the combination of accurate resection of tumor, normal-tissue sparing, and same-day reconstruction under local anesthesia.
